# Supplementary material for: The Systems Biology Research Tool: evolvable open-source software
Source: BMC Syst Biol. 2008 Jun 29;2:55. doi: 10.1186/1752-0509-2-55 (PMC2446383; doi:10.1186/1752-0509-2-55)
Supplement: Additional file 1 — SBRT Archive. An archive of the current version of the Systems Biology Research Tool. [file 1752-0509-2-55-S1.zip › sbrt-1.4.0/doc/developers_guide/api/sbrt/kernel/math/graph_theory/class-use/DirectedGraph.Edge.html]

Uses of Class sbrt.kernel.math.graph\_theory.DirectedGraph.Edge


|  |  |  |
| --- | --- | --- |
| **Overview** \| **Package** \| **Class** \| **Use** \| **Tree** \| **Deprecated** \| **Index** \| **Help** | |  |
| PREV   NEXT | **FRAMES**    **NO FRAMES**     **All Classes** |  |


---


## **Uses of Class sbrt.kernel.math.graph\_theory.DirectedGraph.Edge**

| Packages that use DirectedGraph.Edge | |
| --- | --- |
| **sbrt.kernel.math.graph\_theory** | Provides classes and interfaces for creating objects relevant to graph theory. |
| **sbrt.shell.mng.graph\_theory** | Provides classes and for creating graph theoretical process managers. |
| **sbrt.shell.text.math** | Provides classes and interfaces for defining formats for mathematical objects. |

Uses of DirectedGraph.Edge in sbrt.kernel.math.graph\_theory

| Methods in sbrt.kernel.math.graph\_theory that return types with arguments of type DirectedGraph.Edge | |
| --- | --- |
| `java.util.Set<DirectedGraph.Edge<N>>` | `DirectedGraph.getEdges()`             Returns the set of all edges contained in this graph. |

Constructor parameters in sbrt.kernel.math.graph\_theory with type arguments of type DirectedGraph.Edge

`DirectedGraph(java.util.Set<DirectedGraph.Edge<N>> edges)`
  
          Constructs a new directed graph from the provided
collection of edges.

Uses of DirectedGraph.Edge in sbrt.shell.mng.graph\_theory

| Methods in sbrt.shell.mng.graph\_theory that return types with arguments of type DirectedGraph.Edge | |
| --- | --- |
| `static java.util.Set<DirectedGraph.Edge<java.lang.String>>` | `PathIdManager.getEdges(java.lang.String fileName)`             Parses the file with the provided name, and returns the set of edges it contains. |
| `static <N> java.util.Set<DirectedGraph.Edge<N>>` | `PathIdManager.getEdges(java.lang.String fileName, DirEdgeFormat<N> format)`             Parses the file with the provided name, and returns the set of edges it contains. |

Uses of DirectedGraph.Edge in sbrt.shell.text.math

| Methods in sbrt.shell.text.math that return DirectedGraph.Edge | |
| --- | --- |
| `DirectedGraph.Edge<N>` | `DirEdgeFormatV1.parse(java.lang.String edge)`             Parses the provided string and returns its corresponding directed edge. |

| Methods in sbrt.shell.text.math with parameters of type DirectedGraph.Edge | |
| --- | --- |
| `java.lang.String` | `DirEdgeFormatV1.format(DirectedGraph.Edge<N> edge)`             Returns a formatted string representation of the provided edge. |

---


|  |  |  |
| --- | --- | --- |
| **Overview** \| **Package** \| **Class** \| **Use** \| **Tree** \| **Deprecated** \| **Index** \| **Help** | |  |
| PREV   NEXT | **FRAMES**    **NO FRAMES**     **All Classes** |  |


---
